# The role of *MMP-12* gene polymorphism − 82 A-to-G (rs2276109) in immunopathology of COPD in polish patients: a case control study

**DOI:** 10.1186/s12881-019-0751-9

**Published:** 2019-01-18

**Authors:** Iwona Gilowska, Edyta Majorczyk, Łukasz Kasper, Katarzyna Bogacz, Jan Szczegielniak, Marta Kasper, Jacek Kaczmarski, Aleksandra Skomudek, Marcin Czerwinski, Krzysztof Sładek

**Affiliations:** 1 Institute of Physiotherapy, Faculty of Physical Education and Physiotherapy, Opole University of Technology, Proszkowska street 76, 45-758 Opole, Poland; 2 Second Department of Internal Medicine of Collegium Medicum, Jagiellonian University in Cracow, Skawińska street 8, 31-066 Kraków, Poland; 3 Faculty of Health Sciences, Jagiellonian University Medical College, Michałowskiego street 12, 31-126 Kraków, Poland; 4 Ludwik Hirszfeld Institute of Immunology and Experimental Therapy, Polish Academy of Sciences, Weigla street 12, 53-114 Wrocław, Poland

**Keywords:** COPD, Metalloproteinase 12, Genetics, SNP, ELISA

## Abstract

**Background:**

Major symptoms of chronic obstructive pulmonary disease (COPD) are chronic bronchitis and emphysema leading from lung tissue destruction, that is an effect of an imbalance between metalloproteinases (MMPs) and their tissue inhibitors activity. As potential factor involved in this COPD pathogenesis, MMP-12 is considered. We investigated the role of genetic polymorphism and protein level of MMP-12 in the COPD development among Poles.

**Methods:**

We analyzed − 82 A > G SNP in the promoter region of *MMP-12* gene (rs2276109) among 335 smoked COPD patients and 309 healthy individuals, including 110 smokers. Additionally, 60 COPD patients and 61 controls (23 smokers) were tested for serum levels of MMP-12 using ELISA. All subjects were analyzed for lung function using spirometry (FEV_1_% and FEV_1_/FVC parameters).

**Results:**

We observed that -82G allele and -82GG homozygous genotype frequencies of the SNP rs2276109 were significantly lower in COPD patients than in controls (12.5% vs 16.9%, respectively; χ^2^ = 4.742, *p* = 0.02 for allele and 0.5% vs 3.9%, respectively; χ^2^ = 9.0331, *p* = 0.01 for genotype). Moreover, −82G allele was more frequent in controls smokers than in non-smokers (22.3% vs 14.1%, χ^2^ = 6.7588, p = 0.01). Serum level of MMP-12 was significantly higher in COPD patients than in controls groups (6.8 ng/ml vs 3.3 ng/ml, respectively; F = 7.433, *p* < 0.0001), although independently of analyzed gene polymorphisms. Additionally, no correlation between parameters of lung function (FEV_1_% and FEV_1_/FVC) and protein level was found.

**Conclusions:**

We found that -82G allele of SNP rs2276109 was associated with reduced risk of COPD, and COPD patients released more MMP-12 than healthy individuals, but independently on this SNP.

**Electronic supplementary material:**

The online version of this article (10.1186/s12881-019-0751-9) contains supplementary material, which is available to authorized users.

## Background

Chronic obstructive pulmonary disease (COPD) is a multifactorial disease with gene-environment interaction leading to airflow limitation through the respiratory tract that is not fully reversible [1]. Initially, inflammation of the respiratory tract can protect against the stimuli of harmful gases and dusts, but eventually it results in a chronic inflammatory state [2]. This phenomenon additionally leads to pulmonary emphysema, which is considered, together with chronic bronchitis, as a major symptom of COPD [3]. The emphysema is associated with lung tissue destruction and a loss of lung elasticity, which seem to be the effects of an imbalance in the action of proteases and antiproteases, including metalloproteinases (MMPs) [3]. Due to the capability of degrading matrix proteins (elastin, collagens) MMPs play a crucial role in cell and tissue behaviors such as cell proliferation/migration, angiogenesis, apoptosis, tissues repair and remodeling. This MMP activity is physiologically expected but needs to be controlled primarily by tissue inhibitors of metalloproteinases (TIMPs) that are able to bind to an active site of MMPs, inhibiting their action [4]. However, in pathological conditions such as COPD, chronic inflammation is stimulated by matrix proteolysis-related facilitation of immune cell migration to lung tissues [3].

With regard to above-mentioned MMP function and under the hypothesis of COPD such as protease-antiprotease imbalance, a previous study implicated metalloproteinase 12 (MMP-12) in the pathogenesis of COPD [5]. MMP-12, also called macrophage metalloelastase, is an enzyme released by macrophages that breaks down elastin [6]. It seems to be necessary for emphysema development [7, 8], and specifically, it was shown that elevated levels of MMP-12 in the sputum are associated with emphysema severity in COPD [9–11].

The suggested role of MMP-12 is consistent with the importance of alveolar macrophages (AM) in the pathogenesis of COPD [12]. Macrophages exhibit two distinct phenotypes: M1 producing proinflammatory cytokines, e.g. TNF, IL-6 and IL-12, and M2 with secretion of anti-inflammatory molecules (TGF-β, IL-10) [13]. In COPD it was shown that AM are characterized by both M1 surface phenotypes and functional properties due to the high amount of M1 cytokines, such as TNF and IL-8 [14]. Generally, COPD-related AM produce destructive proteases and their number was ~ 10-fold higher in the lungs of smokers than non-smokers (reviewed by Gharib et al) [15].

Due of this, over-expression of MMP-12 seems to be a result of an increased influx of macrophages, but on the other hand, it was shown that a functional variant in the *MMP-12* promoter (a substitution A-to-G at position − 82; rs2276109) affects the enzyme expression level and COPD susceptibility. It is showed that this substitution influences the binding of the AP-1 (activator protein 1) transcription factor, which possess higher affinity for the -82A allele [16]. As a result, the promoter activity and the MMP-12 expression level may be decrease by allele G, which is associated with a beneficial effect on lung function in children with asthma, as well as a reduced risk for adult smokers to develop COPD [17].

Hence, the aim of this study was to investigate the role of SNP rs2276109 in COPD development in Polish patients and its impact on both a MMP-12 protein level in serum and the lung function spirometry parameters.

## Methods

### Study populations

Three hundred and thirty-five patients (248 males and 87 females) with COPD were enrolled in the study. COPD diagnosis was performed according to the GOLD (Global Initiative for Chronic Obstructive Lung Disease) standards and routinely included the lung function test by spirometry performed twice: before and 15–20 min after bronchodilator application (400 μg of salbutamol). Only patients for whom the bronchial relaxation trial was negative and the post-bronchodilator FEV_1_/FVC ratio was reduced below the lower limit of the norm (< 0.70) were included in the study. In accordance with the GOLD grading system [1], the cases were classified as patients with mild (11% of patients), moderate (49%) and severe/very severe (40%) COPD. Exclusion criteria were alpha-1-antitrypsin deficiency, coexistence of asthma and COPD, history of bronchial asthma and no history of smoking. All patients were recruited from the inpatient and outpatient populations of the University Hospital in Cracow, Specialized Hospital of Ministry of Internal Affairs and Administration (MSWiA) in Głuchołazy and the Department of Pulmonology, Opole Voivodeship Hospital.

In the healthy control group 309 volunteers (80 men and 229 women) were recruited. All subjects were not diagnosed for COPD or any other lung disease and were once spirometrically tested for lung function. Only individuals who exhibited normal lung function were subjected to the test (mean FEV_1_% of 91.7%). Among controls, 110 individuals were defined as smokers (current and former), and 199 participants were never smokers.

The groups were analyzed for single nucleotide polymorphisms, but 60 patients with COPD and 61 control individuals (23 smokers and 38 never smokers) were randomly selected for examination of protein level.

All members of the patients and control group were of Polish Caucasians ethnicity, and detailed characteristics are shown in Table 1.

**Table 1:** Characteristics of the analyzed groups

Groups (N)	Age	Sex	FEV_1_	FEV_1_/FVC	Smokers
	(years)	(women/men)	(% predicted)	(% predicted)	(%)
COPD (335)	67.9 ± 9.2	248/87^b^	55.5 ± 18.5^b^	52.2 ± 16.0^b^	100^b^
CTR (309)	68.0 ± 6.6	80/229	91.7 ± 22.8	89.2 ± 25.8	35.5
CTR smokers (110)	68.4 ± 5.2	73/37	93.3 ± 22.0	91.1 ± 19.5	100
CTR non-smokers (199)	65.8 ± 9.6	157/42	91.0 ± 23.6	88.1 ± 28.7	0
COPD^a^ (60)	70.4 ± 9.0	13/47^b^	46.6 ± 18.5^b^	58.1 ± 16.4^b^	100^b^
CTR^a^ (61)	67.8 ± 6.7	46/15	90.4 ± 22.5	92.6 ± 23.2	37.7
CTR smokers^a^ (23)	67.7 ± 5.6	15/8	93.2 ± 21.9	88.2 ± 16.4	100
CTR non-smokers^a^ (38)	67.9 ± 7.6	31/7	90.6 ± 22.0	90.7 ± 18.0	0

*N* numbers of individuals, *COPD* chronic obstructive pulmonary disease groups, *CTR* control group; ^a^, groups for protein levels analyses, *FEV*_*1*_ forced expiratory volume in 1 s, *FEV*_*1*_*/FVC* Tiffeneau-Pinelli index – ratio of forced expiratory volume in 1 s and forced vital capacity; ^b^, difference to the control group statistically significant at the level of *p* < 0.00001

The project was approved by the Ethics Committee of Opole Voivodship. Signed informed consent was obtained from all persons tested.

### DNA isolation and SNP genotyping

gDNA was extracted from whole blood using the GeneMATRIX Quick Blood DNA Purification Kit (Eurx, Germany) following the manufacturer’s instructions.

The -82A > G polymorphism of the *MMP-12* gene (rs2276109) was genotyped using a TaqMan Genotyping Assay (Applied Biosystems, USA) on a 7500 Fast Real-Time System instrument and SDS 2.0 software (Applied Biosystems) in accordance with the manufacturer’s protocol. Briefly, 10 μl of PCR mixture contained 1x concentrated genotyping master mix, 1x TaqMan Genotyping Assay probes and primer mix, and up to 6.5 ng of DNA. The PCR program was performed under the following conditions: 95 °C, 10 min; 40 cycles of 15 s at 95 °C, 60 s at 60 °C. Allelic discrimination was based on measuring the fluorescence signals generated by two MGB TaqMan probes, one for each allele.

### Analysis of protein level

The serum level of MMP-12 was measured using enzyme-linked immunosorbent assay (ELISA; EIAab, China) accordingly to the manufacturer’s instructions using non-diluted serum samples. The absorbance was read with the Epoch microplate reader (BioTech, Winooski, VT, USA) at the wavelength of 450 nm. Concentrations of proteins in samples were calculated with the equation of the standard curve using Gen5 software (BioTech, Winooski, VT, USA).

### Statistical analysis

Testing of the Hardy–Weinberg equilibrium for the analyzed SNP was performed using the χ^2^ test and EpiInfo software. Case-control differences in alleles and genotypes were estimated using the two-tailed Fisher’s exact test and χ^2^ test with Yates’ correction, respectively and GraphPad InStat 3 software. Moreover, logistic regression with binary response (a disease status) for the multiple inheritance models (co-dominant, dominant, recessive, over-dominant and log-additive) was used (www.snpstats.net). In spirometry data and protein level analyses, the Shapiro-Wilk method was used for testing normality of samples. Relevant statistical comparisons were performed using one-way analysis of variance (ANOVA), and appropriate Tukey post-hoc tests were applied when significant interactions in ANOVA were identified. These statistical analyses were performed with Statistica v.12 (StatSoft, Inc., OK, USA). *P*-values ≤0.05 were considered significant.

## Results

Three hundred and thirty-four patients affected with COPD and three hundred and four healthy individuals were typed for SNP rs2276109 in the gene encoding MMP-12. The observed genotype frequencies were very close to the values expected for both case and control populations according to the Hardy-Weinberg equilibrium (*p* > 0.05, data not shown).

**Fig. 1:**
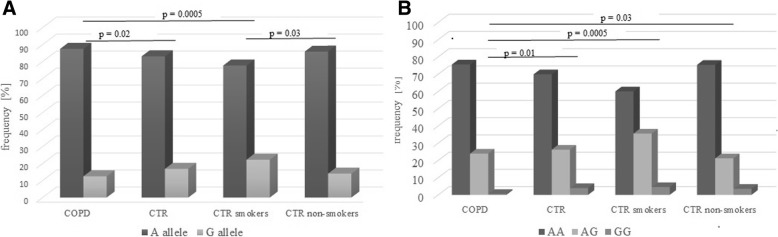
Alleles (**a**) and genotypes (**b**) distribution of − 82 A-to-G SNP of *MMP-12* gene (rs2276109) in COPD patients and healthy control groups. Legend: COPD, patients with chronic obstructive pulmonary disease; CTR – controls; OR (95%CI) calculated for -82G allele in COPD patients-controls comparison was of 0.70 (0.51–0.95)

When allele and genotype frequencies of polymorphism of A-to-G at position − 82 were compared, a significant difference was found. The -82G allele was significantly less frequent in the COPD patients than both in the controls (12.5% vs 16.9%, respectively, *p* = 0.02; OR = 0.70) and in the control smoker group (12.5% vs 22.3%, *p* = 0.0005, OR = 0.50, Fig. 1). In addition, homozygotes for the G allele (GG genotype) were rarer in the COPD group than in the controls (0.5% vs 3.9%, respectively, *p* = 0.01 and OR = 0.14; Fig. 1 and Additional file 1) as well as in control groups divided into two groups accordingly to the smoking status (4.5%, p = 0.0005 and 3.5%, *p* = 0.03 for smokers and non-smokers, respectively). Additionally, the difference between patients with COPD was observed in recessive (AA+AG vs. GG) model of inheritance (see Additional file 1). Moreover, smoker controls significantly differ from non-smokers in -82G allele distribution (22.3% vs 14.1%, p = 0.01). On the other hand, no statistically significant differences in -82A > G allele and genotype frequencies between COPD patients’ groups divided accordingly to the disease severity were found (data not shown).

**Fig. 2:**
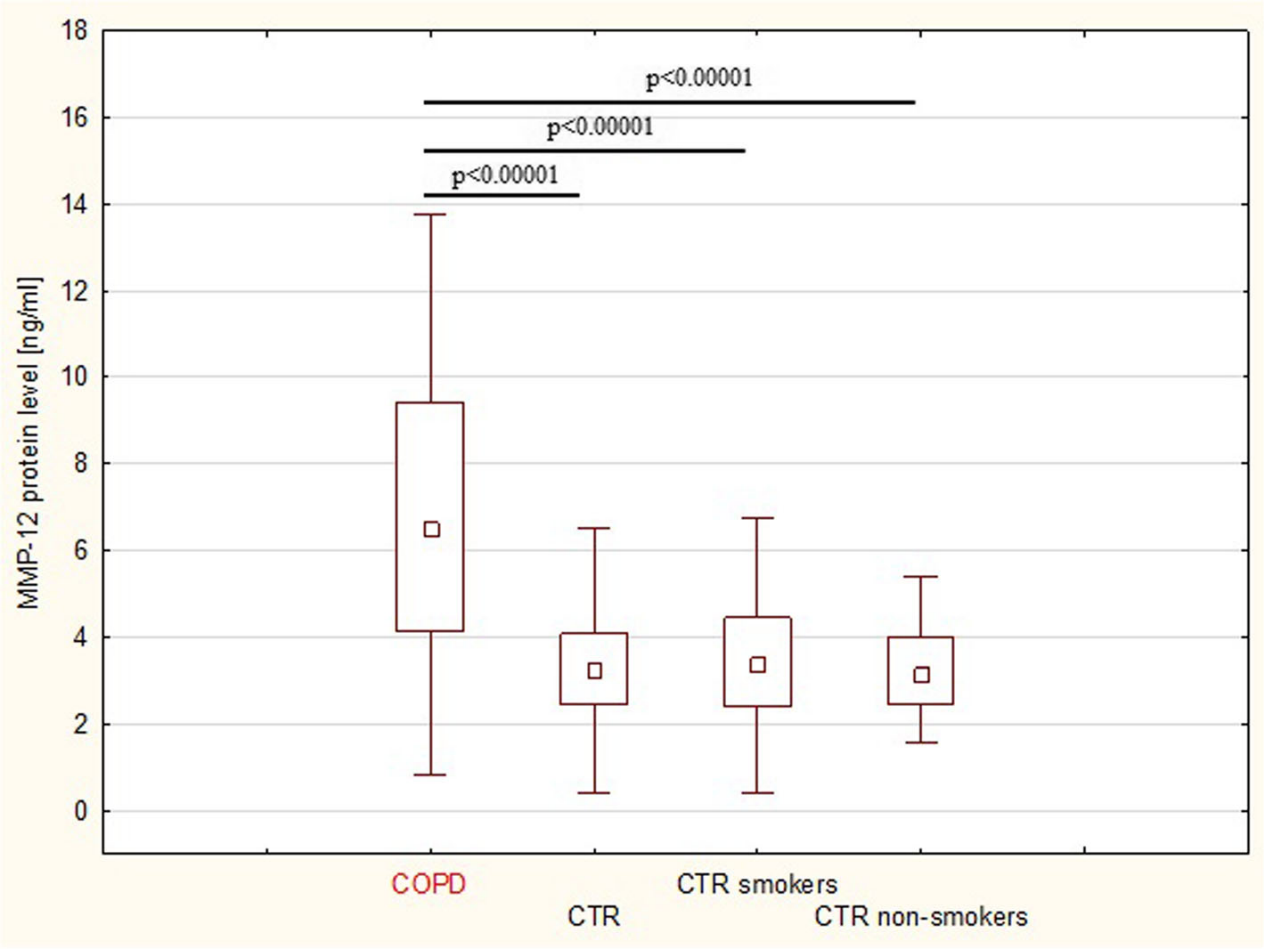
MMP-12 protein level in serum of COPD patients and healthy control groups. Legend: COPD, patients with chronic obstructive pulmonary disease; CTR – controls; data expressed as medians (25 and 75% percentiles)

The serum MMP-12 levels of 60 patients with COPD and 61 age-matched controls were measured by ELISA. As shown in Fig. 2, patients with COPD had significantly higher serum MMP-12 protein levels than controls (6.8 ± 3.3 ng/ml and 3.4 ± 1.3 ng/ml, respectively, *p* < 0.0001). Controls were divided into smoker and non-smoker groups, but similar MMP-12 protein levels were found in both groups (3.2 ± 1.1 and 3.5 ± 1.5 ng/ml, respectively; *p* = 0.45), with significantly lower values compared to patients with COPD (p < 0.0001 for both subgroups).

**Fig. 3:**
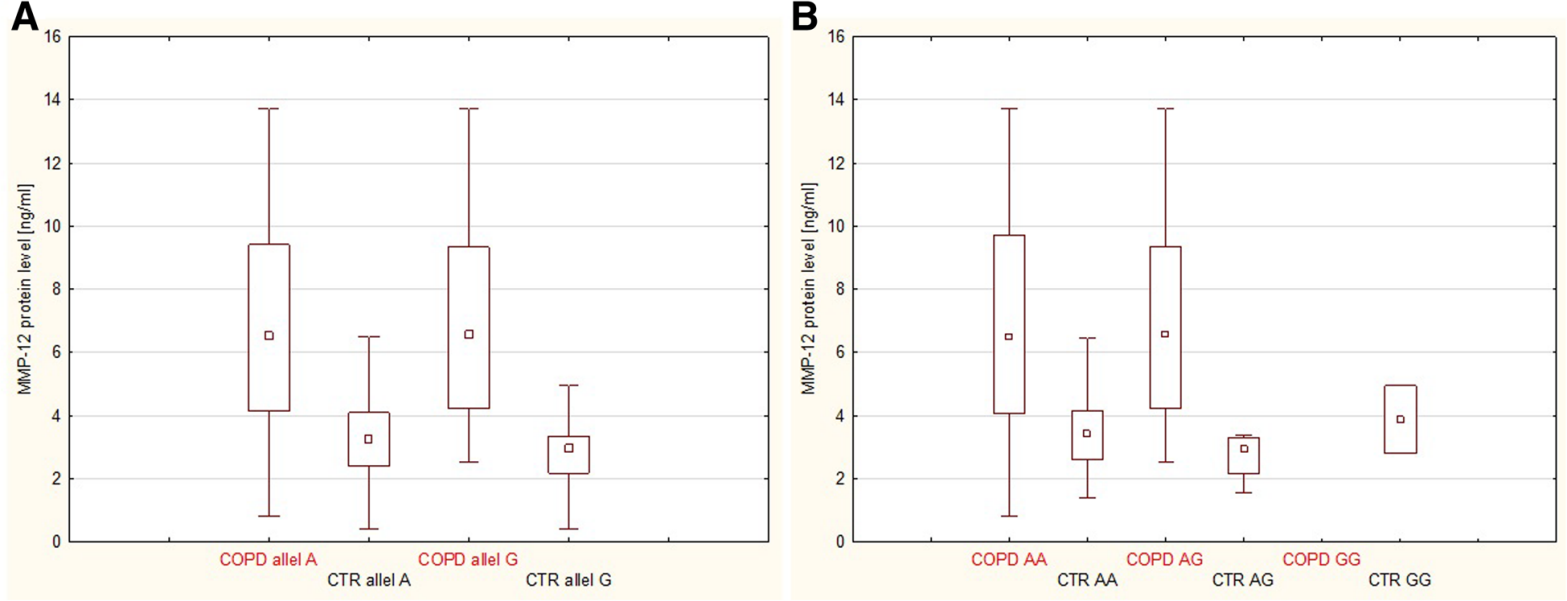
*MMP-12* gene polymorphism −82 A-to-G SNP impact on MMP-12 protein level in serum of COPD patients and healthy control groups (A, allele impact on MMP-12 protein level; B, genotype impact on MMP-12 protein level). Legend: COPD, patients with chronic obstructive pulmonary disease; CTR – controls; data expressed as medians (25 and 75% percentiles); no significant difference in mean MMP-12 protein levels between carriers with different − 82 A > G alleles/genotypes in one-way ANOVA test, Tuckey post-hoc test were not conducted

For the *MMP*-12 -82A > G variants, there were no statistically significant differences in the level of MMP-12 protein among the different genotypes carriers, independently in COPD and control groups (Fig. 3). Moreover, relationships between MMP-12 polymorphism and lung function parameters among both patients with COPD and controls were analyzed. As shown in Table 2, there were no significant relationships between allele/genotypes distribution of − 82 A > G variants and FEV_1_ and FEV_1_/FVC (mean of % predicted).

**Table 2:** *MMP-12* gene polymorphism -82 A-to-G impact on lung function of COPD patients and healthy control groups

Genotypes MMP-12-82A > G	COPD patients			CTR			CTR smokers			CTR non-smokers		
	*N* = 355			*N* = 309			*N* = 110			*N* = 199		
	N	FEV_1_ (% predict.)	FEV_1_/FVC (% predict.)	N	FEV_1_ (% predict.)	FEV_1_/FVC (% predict.)	N	FEV_1_ (% predict.)	FEV_1_/FVC (% predict.)	N	FEV_1_ (% predict.)	FEV_1_/FVC (% predict.)
A allele	333	55.5 ± 18.6	52.3 ± 16.0	297	92.0 ± 23.4	86.2 ± 23.2	105	92.8 ± 21.8	92.0 ± 19.5	192	91.5 ± 23.3	85.8 ± 24.5
G allele	82	54.1 ± 15.8	51.4 ± 17.9	93	92.1 ± 20.7	92.8 ± 24.7	14	94.9 ± 21.9	90.7 ± 20.6	49	88.5 ± 20.4	95.8 ± 27.8
AA	253	55.9 ± 19.3	52.5 ± 15.4	216	92.0 ± 23.4	86.2 ± 23.2	66	92.0 ± 22.2	92.8 ± 18.5	150	91.8 ± 24.2	85.3 ± 26.5
AG	80	53.9 ± 18.0	51.7 ± 18.1	81	92.6 ± 20.1	91.9 ± 21.7	39	94.0 ± 21.4	91.5 ± 21.2	42	90.0 ± 19.9	93.4 ± 22.3
GG	2	56.7 ± 17.4	44.9 ± 18.5	12	88.5 ± 25.3	99.2 ± 40.2	5	101.0 ± 22.6	84.3 ± 15.9	7	79.7 ± 22.3	109.9 ± 47.8
*P* value*		0.71	0.90		0.95	0.10		0.75	0.26		0.30	0.77

*N* number of individuals/alleles/genotypes, *COPD* chronic obstructive pulmonary disease groups, *CTR* control group, *FEV*_*1*_ forced expiratory volume in 1 s, *FEV*_*1*_*/FVC* Tiffeneau-Pinelli index – ratio of forced expiratory volume in 1 s and forced vital capacity; *, no significant difference in mean of lung function parameters between carriers with different −82 A > G alleles/genotypes in one-way ANOVA test, Tuckey post-hoc test were not conducted

## Discussion

In this study, we found that rs2276109 (a substitution A-to-G at position − 82) polymorphism in the *MMP-12* gene can influence susceptibility to COPD in the Polish population, and we confirmed that allele G carriers seem to be protected against the disease. The present study revealed a significant difference in MMP-12 levels between COPD patients and controls. Additionally, no significant relationships were observed between lung function parameters or MMP-12 protein level and the MMP-12 -82A > G polymorphism. To the best of our knowledge, this is the first study to analyze the relationship between rs2276109 and MMP-12 protein level in COPD.

COPD comprises disorders such as chronic bronchitis and emphysema which develop because of air pollution-related low-grade inflammation and destruction of lung tissue, especially extracellular matrix (ECM) [15]. It seems that MMP-12 plays a crucial role in this phenomenon: in a murine model it was shown that the enzyme breaks down the ECM component elastin, and MMP-12-null mice exposed to cigarette smoke avoided emphysema development [7]. In humans, however, as Gharib et al. reviewed, it is very likely that MMP-12 is not effective in direct elastin degradation but could have an impact on this mechanism by affecting macrophage behavior [15].

Macrophages are a cell type possessing dual nature: (i) a major population which patrols lower airspaces in normal conditions, and (ii) predominant inflammatory cells that are recruited in response to cigarette smoke [18]. Due to this, it cannot be excluded that the COPD-related increased level of MMP-12 that was observed in our study is an effect such as stimulated macrophage recruitment in situ. All our patients with COPD were classified as smokers, and it has been previously reported that expression of MMP-12 increased in smokers [10], but this phenomenon was not confirmed in our control smoker and non-smoker subgroups. It could be associated with the finding of greater expression as well as activity of MMP-12 in the sputum of patients with COPD than in smokers without airflow limitation [19].

On the other hand, the enzyme level seems to be related to promoter polymorphism rs2276109. We revealed that a minor allele of SNP rs2276109 seems to protect against COPD development (variant protective effect corresponds with OR value less than 1; Fig. 1). This phenomenon could be explained by functional significance of the -82G allele for decreased activity of the *MMP-12* gene promoter, which is suggested by less efficient binding of transcription factor AP-1 to the G allele [16, 17]. The AP-1 binding region together with STAT-5 is probably critical for induction of MMP-12 expression [20]. Nevertheless, we did not observe a relationship between rs2276109 and MMP-12 protein level, similarly to finding in obesity patients [21]. Therefore, it is most likely that, besides promoter polymorphism, MMPs levels depend on the transcriptional control includes epigenetic mechanisms such as DNA methylation and histone acetylation, and post-transcriptional regulation is by cytosolic mRNA stability [22]. Final MMP-12 concentration in blood seems to be a results of this rigorous control system as well as e.g. number of the cells which secrete the enzyme. On the other hand, in this study, no significant correlation was found between this variant and lung function parameters of COPD, similarly to previous studies [5, 23, 24]. This result (Table 2), however, contrasts with a finding that this variant (−82G) was associated with increased FEV1 and delayed onset of the disease together with the abovementioned reduced risk of COPD [17].

Several limitations of the present study should be taken into account. We measured MMP-12 only at the protein level without mRNA expression level analysis. Second, the protein level was measured in blood, but not in situ using either sputum or bronchoalveolar lavage, which should better reflect the MMP-12 as well as macrophage phenomenon occurring in the COPD-related lung microenvironment. Unfortunately, data about numbers of macrophages and other immunocompetent cells (e.g. neutrophils) were also unavailable in our study. The mentioned limitations were related to the samples gathering and missing data seem to be important for consideration in further research.

## Conclusion

We confirm that patients with COPD exhibited more MMP-12 protein, and the -82G allele of SNP rs2276109 was more frequent in controls than in COPD patients group. On the other hand, no relationship between the mentioned genetic polymorphism and MMP-12 level was found. Nevertheless, our data, together with other findings, suggest that MMP-12 affects COPD development. In our opinion, the molecule could be deliberated in further research as a useful molecular marker as well as therapy target in COPD management.

## Additional file

Additional file 1: **Table 1S.** Logistic regression analysis of association between -82 A-to-G SNP of *MMP12* gene (rs2276109) and COPD – the multiple inheritance models. Description of data: This table contains the logistic regression results of modeled association between SNP rs2276109 of *MMP12* gene and COPD. (DOCX 16 kb)

## Abbreviations

AM: alveolar macrophage
AP-1: activator protein 1
COPD: chronic obstructive pulmonary disease
ECM: extracellular matrix
FEV1: forced expiratory volume in 1 s
FVC: forced vital capacity
GOLD: Global Initiative for Chronic Obstructive Lung Disease
MMP: metalloproteinase
SNP: single nucleotide polymorphism
