# Supplementary material for: The role of MMP-12 gene polymorphism − 82 A-to-G (rs2276109) in immunopathology of COPD in polish patients: a case control study
Source: BMC Med Genet. 2019 Jan 18;20:19. doi: 10.1186/s12881-019-0751-9 (PMC6339316; doi:10.1186/s12881-019-0751-9)
Supplement: Supplementary file 1 — Table 1S. Logistic regression analysis of association between -82 A-to-G SNP of MMP12 gene (rs2276109) and COPD – the multiple inheritance models. Description of data: This table contains the logistic regression results of modeled association between SNP rs2276109 of MMP12 gene and COPD. (DOCX 16 kb) [file 12881_2019_751_MOESM1_ESM.docx]

Additional file 1: Table S1. Logistic regression analysis of association between -82A/G SNP of *MMP12* gene (rs2276109) and COPD – the multiple inheritance models

| Model | rs2276109  genotypes | Frequency (Number of positive) | | COPD vs. CTR | | |
| --- | --- | --- | --- | --- | --- | --- |
|  |  | COPD  N = 335 | CTR  N = 309 |  |  |  |
|  |  |  |  | *P* | OR | 95%CI |
| Codominant | AA | 75.5 (253) | 69.9 (216) | 0.01 | 1.00 | - |
|  | AG | 23.9 (80) | 26.2 (81) |  | 0.84 | 0.59-1.21 |
|  | GG | 0.6 (2) | 3.9 (12) |  | 0.14 | 0.03-0.64 |
| Dominant | AA | 75.5 (253) | 69.9 (216) | 0.11 | 1.00 | - |
|  | AG+GG | 24.5 (82) | 30.1 (93) |  | 0.75 | 0.53-1.07 |
| Recessive | AA+AG | 99.4 (333) | 96.1 (297) | 0.003 | 1.00 | - |
|  | GG | 0.6 (2) | 3.9 (12) |  | 0.15 | 0.03-0.67 |
| Over-dominant | AA+GG | 76.1 (255) | 73.8 (228) | 0.49 | 1.00 | - |
|  | AG | 23.9 (80) | 26.2 (81) |  | 0.88 | 0.62-1.26 |
| Log-additive |  |  |  | 0.02 | 0.70 | 0.51-0.96 |

N, number of individuals; COPD, chronic obstructive pulmonary disease groups; CTR, control group
